# Dissolved Carbon Dioxide Sensing Platform for Freshwater and Saline Water Applications: Characterization and Validation in Aquaculture Environments

**DOI:** 10.3390/s19245513

**Published:** 2019-12-13

**Authors:** J.P. Mendes, L. Coelho, B. Kovacs, J.M.M.M. de Almeida, C.M. Pereira, P.A.S. Jorge, M.T. Borges

**Affiliations:** 1 CIQUP-Chemistry Research Unit, University of Porto, 4169-007 Porto, Portugal; joaomendes.quimica@gmail.com (J.P.M.);; 2 Department of Chemistry and Biochemistry, Faculty of Sciences, University of Porto, 4169-007 Porto, Portugal; 3 INESCTEC-Institute for Systems and Computer Engineering, Technology and Science, 4200-465 Porto, Portugal; 4 CIIMAR—Interdisciplinary Centre of Marine and Environmental Research, 4450-208 Matosinhos, Portugal; 5 Department of General and Physical Chemistry, Faculty of Natural Sciences, University of Pécs, H-7624 Pécs, Hungary; kovacs1@gamma.ttk.pte.hu; 6 Department of Physics, School of Sciences and Technology, University of Trás-os-Montes e Alto Douro, 5001-801 Vila Real, Portugal; 7 Department of Physics and Astronomy, Faculty of Sciences, University of Porto, 4169-007 Porto, Portugal; 8 Department of Biology, Faculty of Sciences, University of Porto, 4169-007 Porto, Portugal

**Keywords:** optical sensor, chemical optrode, dissolved carbon dioxide, aquaculture, optical fiber, colorimetric sensor

## Abstract

A sensing configuration for the real-time monitoring, detection, and quantification of dissolved carbon dioxide (dCO_2_) was developed for aquaculture and other applications in freshwater and saline water. A chemical sensing membrane, based on a colorimetric indicator, is combined with multimode optical fiber and a dual wavelength light-emitting diode (LED) to measure the dCO_2_-induced absorbance changes in a self-referenced ratiometric scheme. The detection and processing were achieved with an embeded solution having a mini spectrometer and microcontroller. For optrode calibration, chemical standard solutions using sodium carbonate in acid media were used. Preliminary results in a laboratory environment showed sensitivity for small added amounts of CO_2_ (0.25 mg·L^−1^). Accuracy and response time were not affected by the type of solution, while precision was affected by salinity. Calibration in freshwater showed a limit of detection (LOD) and a limit of quantification (LOQ) of 1.23 and 1.87 mg·L^−1^, respectively. Results in saline water (2.5%) showed a LOD and LOQ of 1.05 and 1.16 mg·L^−1^, respectively. Generally, performance was improved when moving from fresh to saline water. Studies on the dynamics of dissolved CO_2_ in a recirculating shallow raceway system (SRS+RAS) prototype showed higher precision than the tested commercial sensor. The new sensor is a compact and robust device, and unlike other sensors used in aquaculture, stirring is not required for correct and fast detection. Tests performed showed that this new sensor has a fast accurate detection as well as a strong potential for assessing dCO_2_ dynamics in aquaculture applications.

## 1. Introduction

Dissolved carbon dioxide (herein noted as dCO_2_) is a very important parameter in many different fields, e.g., monitoring oceans, rivers, streams, and lakes, food industry (including aquaculture), and clinical analysis [1,2,3,4,5,6,7,8]. Besides the actual concern with ocean acidification due to the increasing atmospheric carbon dioxide (CO_2_) levels [9,10], dCO_2_ evaluation and control is particularly vital in aquaculture, as cultivated species are highly sensitive to the excess of this gas [11]. A detailed knowledge on short-time dCO_2_ variations in intensive fish production systems (as are recirculating aquaculture systems, RAS) has been hindered, among other factors, by the long response time of the commercial sensors that are in use, as they do not allow highly frequent measurements. Moreover, this parameter can change rapidly with normal fish farm operations (e.g., feeding events, which increased fish and bacterial metabolism and thus dCO_2_ production), or with performance changes of the recirculated water treatment components, such as the biofilter, gassing (O_2_), or degassing (CO_2_) units, which are more pronounced in seawater [12]. So, sensors are needed with fast response time to obtain more dCO_2_ data per unit of time, which is crucial in studies of dynamic systems, as are the recirculating aquaculture systems [13]. Besides fast response time, high sensitivity is another aspect of interest in intensive aquaculture, not only to assess irregularities in system functioning, but also to monitor dCO_2_ concentrations in the very low range (e.g., 1 mg·L^−1^), which are expected, for example, in hatchery and nursery production facilities [13]. Nevertheless, dissolved CO_2_ detection is difficult because when CO_2_ dissolves in water, physical and chemical equilibria occur, and different products are generated [8]. Moreover, commercial sensors for dCO_2_ detection and evaluation in aquaculture environments are scarce, and adequate sensors for more demanding operations, such as real-time monitoring or low operation ranges, are missing [14]. So far, known methods for dCO_2_ measurements in an aqueous medium, include gas chromatography [8,15], colorimetric sensors (fluorescence) [8,16], amperometry [17], potentiometry [4,18,19], UV/Vis spectrophotometry [20,21], and IR spectrometry [8,22]. However, it is reported that evaluating dCO_2_ using some of the techniques referred above is difficult or not adequate for field work, and other analytical techniques are preferred [8]. In aquaculture, the utilization of commercial dCO_2_ analyzer (such as OxyGuard, model Portable CO_2_ Analyzer, Denmark) has been thoroughly studied and described based on dCO_2_ partial pressure detection via IR absorption, needing a certain flow under the probe membrane for best performance [12,14,23]. More recently, optical sensors gained strength in the sensing area and for a wide variety of analytes, with no exception for dissolved carbon dioxide. These sensors can be more robust, compact, fast responding, and cheaper [8,24]. Usually, these optical devices are fluorescence-based (light emission) or colorimetric-based (light absorbance) sensors [8]. An example of the first method utilization is referred by Atamanchuk et al., who used an optrode for measuring the partial pressure of dCO_2_ in natural waters. The sensor is based on pH changes originated by CO_2_ diffusion and fluorescence signal response [25]. Contreras-Gutierrez et al. also developed a dCO_2_ fiber optic sensor using a polymer matrix directly combining a fluorescent dye with the polymer molecule [26]. More recently, Thomas et al. added a second dye to extend the fluorescence lifetime from nanoseconds to microseconds, which reduced the cost of the interrogation system used to interrogate only the fluorescence of the first dye [27]. Normally, these are called “wet sensors” [8]. These kind of sensors require the use of an aqueous solution phase, which can be seen as a disadvantage [25]. Its response can be affected if the osmotic pressure of the tested system is significantly different from that of the sensor [8,28]. On the other hand, it is possible to detect and evaluate dCO_2_ using also the so-called “dry sensors” [8]. This type of sensors use a solid-state system that itself uses a quaternary ammonium, QA^+^OH, as a transfer agent (TA) to detect dissolved carbon dioxide, and these form a very popular system these days [8]. However, it is crucial to choose a suitable material according to the required applications. For example, Mills and Yusufu (2016) [1] reported a promising colorimetric-based dry sensor using a pH-sensitive dye, thymol blue. Nevertheless, the thymol blue membrane decreases its sensibility in water, which can be a problem for applications requiring high sensitivity. Therefore, a material is required that keeps its sensing properties in different application environments. In the present work, a new sensing device was developed, combining a solid-state membrane based on a colorimetric indicator, poly *p*-nitrophenol (pNPh), with multimode fiber optic patch cords and a dual wavelength LED. This sensing platform works with a ratiometric detection scheme to measure the absorbance changes caused by the dCO_2_ interactions with the chemical membrane. The sensing chemistry comprises a thin layer formed by a colorimetric indicator/solvent/TA/plasticizer generated by spin-coating on the top of a Mylar foil (as substrate). The resultant layer contains a sensitive dye in its deprotonated anionic form, which is subsequently covered by a hydrophobic silicone layer fixed in a proper support specially designed for the purpose. The sensor was tested for low dissolved carbon dioxide concentrations (needed for adequate water quality monitoring and control) with the goal of studying its viability for aquaculture applications.

## 2. Materials and Methods

### 2.1. Chemical Reagents and Other Materials

The sensing membrane was obtained through complex cocktails developed with the following chemicals: p-nitrophenol (Sigma-Aldrich ReagentPlus^®^, ≥99%, St. Louis, MO, USA) as a colorimetric monomer for poly pNPh synthesis; tetraoctylammonium hydroxide solution (TOA-OH, Sigma-Aldrich, 20% in methanol) as a quaternary ammonium, and hydrogel D4 solution (AdvanceSource Biochemicals, 10% in Ethanol, 96%; Lawrenceville, NJ, USA). Sylgard 184 (Dow Corning, 10:1; Midland, MI, USA) was used as a permeable silicone to cover the sensing membrane. Dry nitrogen (N_2_) was supplied from a 50 L bottle (Linde, ≥99.99%; Dublin, Ireland) and CO_2_ was supplied from a 30 L bottle (Linde, ≥99.99%). For chemical calibrations, citric acid (C_6_H_8_O_7_, Merck^®^; Darmestadt, Germany) and sodium carbonate (Na_2_CO_3,_ Sigma-Aldrich, >99.5%; St. Louis, MO, USA) were used.

### 2.2. Sensing Layer Preparation and Sensing Chemistry

**Figure 1:**
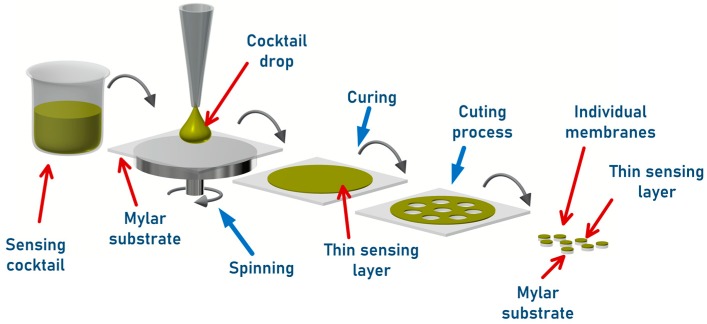
Sensing layer preparation process.

The sensing layers were attained by the dissolution of 2.00 mg·L^−1^ of poly pNPh in 0.05 mL of a MeOH:H_2_O solution (1.5:1) and 0.50 mL of 0.50 M TOA-OH solution. After total dissolution, 0.10 mL of a 10% hydrogel D_4_ solution was added to ensure the formation of a network chain of a single polymer molecule to form one big molecule on the macroscopic scale. The resulting cocktail was spread on a Mylar foil by spin-coating (900 rpm for 60 s) and allowed to dry for 2 h. The resulting sensing membranes should be stored at 4 ºC until the encapsulation process. The sensing chemistry comprises a sensitive dye in its deprotonated anionic form, resulting from the ion pair formation, QA^+^pNPh^−^. The formed ion pair is normally associated with a few molecules of water, QA^+^pNPh^−^.xH_2_O, which can explain its interaction with the dissolved CO_2_ (Equation (1) adapted from reference [8]). Figure 1 shows the preparation process of the sensing membrane.

(1) $$QA^{+}pNPh^{-}\cdot xH_{2}O+CO_{2}⇆QA^{+}HCO_{3}^{-}\cdot \left(x-1\right)H_{2}O\cdot HpNPh$$

### 2.3. Dissolved CO_2_ Optrode Configuration

**Figure 2:**
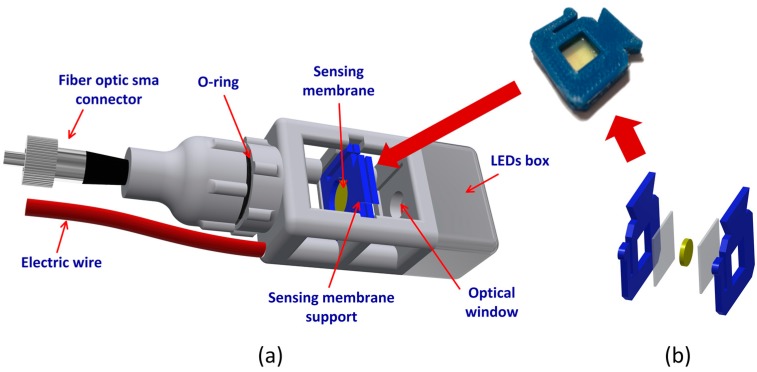
(**a**) Sensing head assemble and sensing layer position; (**b**) Sensing layer encapsulation.

The sensing device is based on a transmission colorimetric setup combining a sensing layer placed between a dual wavelength LED and a large core optical fiber (Figure 2a).

As mentioned before, the sensing layer needs to be encapsulated between two protective membranes, allowing it to be dipped in water. For this process, two plastic supports covered by a thin film (approximately 90 µm) of Sylgard 184 (base:curing agent, 10:1; Dow Corning, Midland, MI, USA ), previously cured, were used. The sensing membrane was placed between the plastic parts that were sealed with fresh silicone and allowed to dry overnight at 25 °C. The sensing membrane support (Figure 2b) and the sensing head structure (Figure 2a) were 3D printed (Zortax M2000; material: Z-Ultra T; Olsztyn, Poland). As shown in Figure 2a, the sensing head is composed by a light-emitting diode (LED) box containing an additive color model in which red, green, and blue light are added together in various ways to reproduce a broad array of colors (a.k.a. RGB LED; Kingbrigth Europe, Lindenau, Issum, Germany), powered by an electric wire, working in two specific wavelengths: 460 nm for blue light (detection band) and 645 nm for red light (reference signal). A fiber optic connector was placed in the other extreme to attach a fiber optic cable to guide the light to a mini spectrometer (Hamamatsu C12880MA, Shizuoka, Japan) in the UV-Vis band incorporated into an electronic platform specially designed for this purpose. Both sides of the sensing head are separated from the external medium by an optical window (glass) that also contains two plastic lenses (Roithner LaserTechnik GmbH, Wien, Austria) inside to collimate the beam, which improves the collected signal. This configuration allows a great proximity (approximately 100 µm distance) between the aqueous medium and the sensing surface, which measures without stirring (similarly to potentiometric electrochemical sensors and some optrodes). The use of fiber optic cables allows for potentially real time, multipoint, and continuous measurements as well as the construction of a miniaturized sensing platform [14,29]. As the mini spectrometer needs to be connected to a PC, the data acquisition is possible at a larger scale. For data acquisition and real-time data observation, homemade LabView interactive software was designed to process the optical signals.

### 2.4. Assessment of the dCO_2_ Optrode Performance

#### 2.4.1. Experimental Setup and Conditions for Laboratory Assays

**Figure 3:**
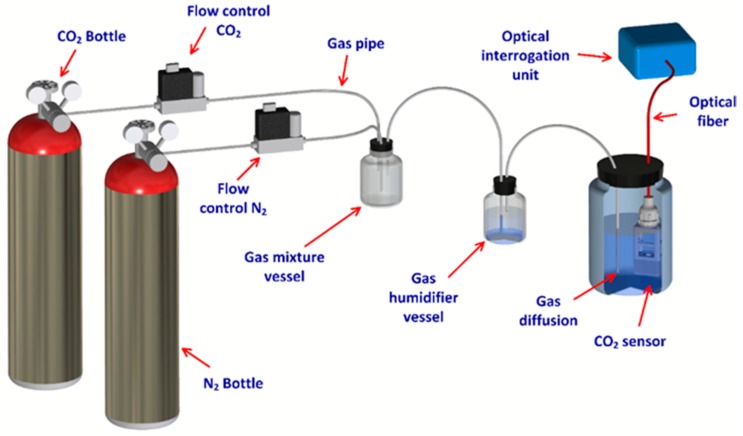
Schematic assemblage of the CO_2_/dCO_2_ (dissolved carbon dioxide) new sensor evaluation tests.

A set of tests was performed to evaluate the optrode performance in different laboratory situations. Initially, the sensor was tested with a humidified gas mixture composed by CO_2_ and N_2_ supplied by gas bottles. The percentage of the gases in the mixture was varied using high-resolution mass-flow controllers (Brooks, SLA5800 Series; Hatfield, PA, USA)). The gases were mixed in a vessel before humidification. These types of tests were performed to confirm that the chemical membrane was sensitive to gaseous CO_2_ variations. Further laboratory assays were conducted using the same gas mixing bottle with 200 mL of deionized water (Wasserlab, Micromatic-Type II Analytical Grade Water; Barbatáin (Navarra), Spain). The same concentrations of gases were injected to test the sensor behavior to dCO_2_ variations. Both tests were made without stirring. Figure 3 shows the general scheme of the laboratory setup.

#### 2.4.2. Optrode Calibration

The sensor was calibrated using deionized water and saline solution (at 2.5%) for a range of dCO_2_ concentrations from 1.00 to 20.00 mg·L^−1^. The maximum value tested was the maximum recommended value for fish farming [30]. The calibration tests were made using a standard Na_2_CO_3_ solution (0.300 ± 0.001 g dissolved in 50.00 ± 0.05 mL of ultra-pure water) with well-known dCO_2_ concentrations in deionized water and in saline aqueous solution (2.5%), which were previously acidified with citric acid (pH ≈ 3; V = 200 mL). The formation of carbon dioxide results from the stoichiometric reaction of sodium carbonate in an aqueous citric acid solution, as shown in Equation (2). This procedure was adopted in order to compare the results with calibration procedures implemented by commercial CO_2_ analyzers (namely OxyGuard).

(2) $$3CO_{3}^{2-}+2C_{6}H_{8}O_{7}\to 2Na_{3}{\left(C_{6}H_{5}O_{7}\right)}^{3-}+3H_{2}O+3CO_{2}$$

Through the calibration curve it was possible to determine the sensor limit of detection (LOD) and the limit of quantification (LOQ) using the “calibration curve parameters method” [31]. The mathematical Equations (3) and (4) were used for LOD calculation, where *Y_LD_* is the instrumental signal of the LOD, *a*_0_ is the zero intercept value, *t* is the unilateral critical value for a 90% confidence interval for (n-p) degrees of freedom (*n* is the number of points of the calibration curve; *p* is the number of parameters), *SDa*_0_ is the standard deviation of the zero intercept value, *X_LD_* is the concentration corresponding to the LOD instrumental signal, and *b* is the slope of the calibration curve. Equations (5) and (6) were used for LOQ, where *Y_LQ_* is the instrumental signal of the LOQ, and *X_LQ_* is the concentration corresponding to the LOQ instrumental signal (adapted from reference 30). All the other symbols for Equations (5) and (6) are described in the description of Equations (3) and (4).

(3) $$y_{LD}=a_{0}+2\times t_{0.10\left(n-p\right)}^{u}\times SD_{a_{0}}$$

(4) $$x_{LD}=\frac{y_{LD}-a_{0}}{b}$$

(5) $$y_{LQ}=a_{0}+3\times 2\times t_{0.10\left(n-p\right)}^{u}\times SD_{a_{0}}$$

(6) $$x_{LQ}=\frac{y_{LQ}-a_{0}}{b}$$

#### 2.4.3. Optrode Precision, Accuracy, Sensitivity and Response Time in Deionized Water and Saline Water (2.5%)

The calibration procedure was followed by sequential tests to evaluate precision (in the form of coefficient of variation; CV, % = standard deviation (SD)/mean × 100), accuracy ((real value − experimental value)/real value), and response time (signal time stabilization to 95% of equilibrium response, *t*_95%_) in the following water conditions: deionized water and saline water (2.5%). Different dCO_2_ concentrations were tested in the range from 1.00 to 20.00 mg·L^−1^. Three independent runs were done for each concentration, and the data acquisition was made every 100 milliseconds. As the response time, it was considered the time taken by the sensor to reach a new concentration starting from the immediately preceding concentration (cumulative time). With the goal of comparing the new sensor response time with the response time of a commercial sensor recommended for aquaculture (OxyGuard CO_2_ Analyzer^®^ with data logging; OxyGuard, Farum, Denmark), tests using the two different devices were made in deionized water. Optrode sensitivity (ability to detect small variations) in a very low dCO_2_ range was also tested in the same water conditions. Known dCO_2_ concentrations were varied by the incremental addition of 0.25 mg·L^−1^ between 1.00 and 2.00 mg·L^−1^ until total signal stabilization. For this study, standard solutions were used as described in Section 2.4.2.

#### 2.4.4. Agitation Conditions

All the tests performed with the new sensor using gas injection were made without agitation, including the dissolved CO_2_ dynamics studies. However, some agitation was required for the calibration curve measurements to promote the reaction between the known amount of solid Na_2_CO_3_ added to the acid media and produce the expected amounts of dCO_2_. Laboratory tests using the OxyGuard analyzer were always performed with stirring.

### 2.5. Dissolved CO_2_ Dynamics in a Recirculating Shallow Raceway System (SRS+RAS) Prototype: Performance Comparison between New dCO_2_ Optrode and Oxyguard CO_2_ Analyzer^®^

#### 2.5.1. Prototype Characteristics

One of the aims of this work was to contribute to a solution for the dCO_2_ monitoring needs of the aquaculture industry. Nevertheless, when working in real aquaculture facilities, several uncontrolled events occur, which need to be avoided to guarantee consistent testing and the monitoring of dCO_2_ in water. This can only be achieved in controlled laboratory conditions. Therefore, to be able to simulate diverse intensive aquaculture situations, a laboratory prototype was needed. So, a shallow raceway tank (SRS) was used with 0.4 m^2^ of area and 7 cm of water height, as reported by Borges et al., which was linked to a water treatment loop (with sump, mechanical filter, and aerated biological filter), representing a total working volume of 100 L [14]. The whole system mimicked a recirculating aquaculture system (RAS) working at >95% water recycling.

#### 2.5.2. Dissolved CO_2_ Dynamics Studies in the SRS+RAS Prototype

Gas injection was done using a special cylindrical diffuser (Dupla CO_2_ reactor 400), which was connected to an Eheim Hj-731, 550 L·h^−1^ pump. Carbon dioxide was delivered by an aquarium-type compressed CO_2_ system (Dupla CO_2_ Set Delta 400). The injection device was placed near the bottom of the tank, after the inlet pipe. The continuous monitoring of dCO_2_ was made using the new dCO_2_ optrode as well as the OxyGuard CO_2_ Analyzer^®^ with logging capabilities. Both devices were placed near of the prototype tank outlet. Two independent runs were done, injecting gaseous CO_2_ for 1 h and 15 min with a flow rate of approximately 10 mL·min^−1^. The next run was carried out after complete CO_2_ degassing, as indicated by zero readings in the sensors. In both tests, trout food was introduced into the water, and after some time, the excess was removed to simulate the fish feeding process and study the behavior of the new sensor under these conditions. The experiments were performed in a controlled temperature room at 15 °C, using dechlorinated tap water.

### 2.6. Continuous dCO_2_ Monitoring in an Experimental Fish Culture System with Water Recirculation

Short-time monitoring of a fish culture system was performed in a laboratory fish culture system using water recirculation in order to evaluate the behavior of the sensor under in situ conditions and continuous dissolved CO_2_ monitoring. The aquaculture recirculation system consisted of two fish tanks of 300 L each and contained a mechanical filter, a sump tank with a trickling filter, and a third tank equipped with a moving bed biofilter (MBBR). The system, with a total volume of 1000 L of freshwater, was operated at 80% RAS. Cultivated fish were rainbow trout (*Oncorhynchus mykiss*), with 110 g average weight and a density of 10.6 kg.m^−3^. Sampling points comprehended one fish tank and the tank containing the MBBR biofilter. The new dCO_2_ optrode and the commercial sensor Oxyguard CO_2_ Analyzer^®^ were used simultaneously for dCO_2_ evaluation.

## 3. Results

### 3.1. Experimental Laboratory Results: Gaseous CO_2_ and dCO_2_ Measurements

**Figure 4:**
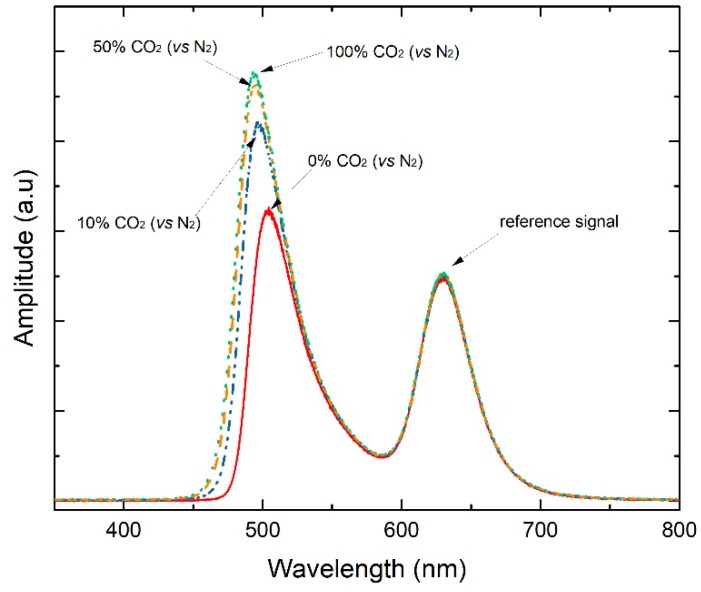
Response of the new optrode to gaseous CO_2_ variations to test the activity of the sensing membrane.

The sensing membrane response was evaluated in a closed container with a controlled gas mixture of CO_2_ and N_2_ to simulate different concentrations of CO_2_. Some of the obtained spectra are shown in Figure 4. As expected, the obtained signal for red light did not change its amplitude, while the blue LED signal enhanced its amplitude with the CO_2_ increase. This signal enhancement is associated to a color intensity decrease by the membrane due to its reaction with CO_2_. Comparing the relative change of the intensity peaks at each gaseous CO_2_ concentrations, it was demonstrated that the new sensor has higher sensitivity for the lower CO_2_ concentrations tested.

**Figure 5:**
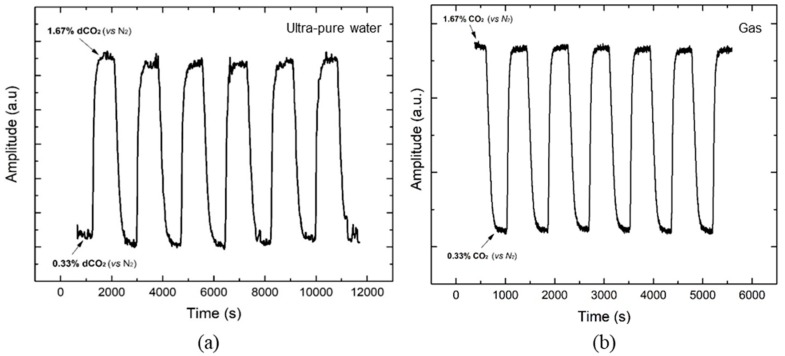
Obtained timelines for stability tests in (**a**) pure water and in (**b**) gas environment.

At this point, it was notorious that the sensing membrane is sensible to CO_2_ variations and the sensor was ready to be tested in aqueous media. Thus, it was required for the membrane to be waterproof. This was a difficult and lengthy process, and several approaches were tried to encapsulate the sensing membrane. The first method attempted consisted of spin coating the used silicone directly on the top of the sensing membrane [32]. However, some reactions between the sensing layer and the silicone were observed, which affected the silicone-curing process. A second attempt used the “knife technique”. At this phase, the silicone was spread on the top of the sensing layer using a spatula, controlling the silicone layer thickness using mechanical guides with known height. As the problems reported before were also verified, a new approach was needed. So, the solution was spin coating the silicone on the top of a substrate (Mylar foil) and forming a cured silicone porous membrane that was later on attached onto the sensing membrane using specially designed 3D-printed supports. To study the sensor stability in water, a glass container with 200 mL of deionized water was used where different dCO_2_ concentrations (in percentage versus N_2_) were bubbled. Several cycles were done between 0.33% and 1.67% of dCO_2_ (always keeping the same flow rate)_,_ during more than 3 h. A timeline graph was plotted, which is shown in Figure 5a. The stability of the sensor was confirmed. However, some fluctuations are visible when the sensor achieves the target concentration. These variations can be explained by the turbulence produced by the bubbles of gas in water. To compare the behavior of the sensor in the two environments (dCO_2_ versus CO_2_), a test in gas atmosphere, using humidified air, was performed. The resultant timeline can be seen in Figure 5b. A total of six complete cycles were done in each experiment, and it is possible to see that in air, the six cycles were completed in 1 h and 40 min against the 3 h and 20 min of the test in water. This increase in the signal response time is expected due to the time that the gases take to dissolve in water. The resistance offered by the water to the degassing from the membrane also contributes to the increase in the signal response time. As the CO_2_ in the system was gas injected in this test, the time observed cannot be measured as the sensor response time and should be considered the setup response time. Moreover, the sensor response time was estimated using a supersaturated standard solution in Section 3.3.

### 3.2. Optrode Calibration Curves and Associated Data

**Figure 6:**
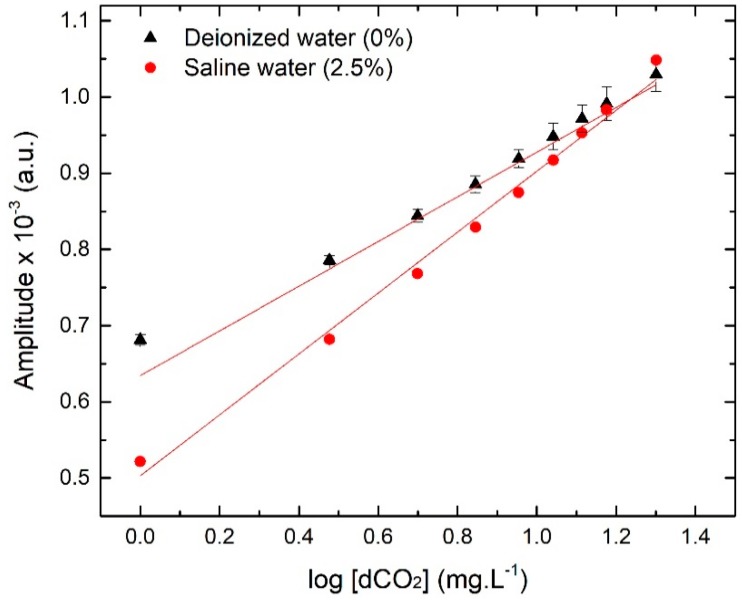
New dCO_2_ sensor calibration curves plotted as a function of dCO_2_ logarithmic concentration in different aqueous solutions.

The sensor was calibrated for an operation range between 1.00 and 20.00 mg·L^−1^dCO_2_ in different aqueous solutions (deionized water and saline water at 2.5%). As referred before, this is the relevant concentration range for aquaculture operations. Calibration curves and calibration data can be found in Figure 6 and Table 1, respectively. The sensor response to the successive standard dCO_2_ solutions is logarithmic, so the data were plotted as a function of dCO_2_ logarithmic concentration to attain linearity. The results show a different sensor response for fresh and saline water. This interfering process resulted in unexpected results obtained for the calibration curve in saline water, which should run in the complete concentration range above the curve for deionized water [33]. Eventually, the presence of salt is affecting the silicone membrane permeability and interfering with the calibration process. The understanding of this process needs more extensive study.

The LODs and LOQs (Table 1) are also different for the different aqueous solutions. Nevertheless, these results suggest that the sensor is suitable to work in the concentration range found in aquaculture for both types of water [34].

### 3.3. Optrode Precision, Accuracy, and Response Time in Deionized Water and Saline Water (2.5%)

The new dCO_2_ optrode performance data for a range of concentrations between 1.00 and 19.8 mg·L^−1^ in different aqueous solutions can be found in Table 2. There are different ranges for precision in the different aqueous solutions. For deionized water, the range of precision is from 5.87% to 19.1%, while for saline water (2.5%), the range of precision is from 1.19% to 1.43%. These results show that the new sensor is more precise for measurements in saline water. It is possible to see that the precision in both cases is better in the range from 1.00 to 8.97 mg·L^−1^, which is the less dangerous range to fish life and that which is normally is found in aquaculture waters. The accuracy changed from the extreme concentrations (1.00 and 19.8 mg·L^−1^) to the intermediate concentrations: 2.99 to 12.9 mg·L^−1^ for deionized water and 2.99 to 14.9 mg·L^−1^ for saline water (2.5%).

**Figure 7:**
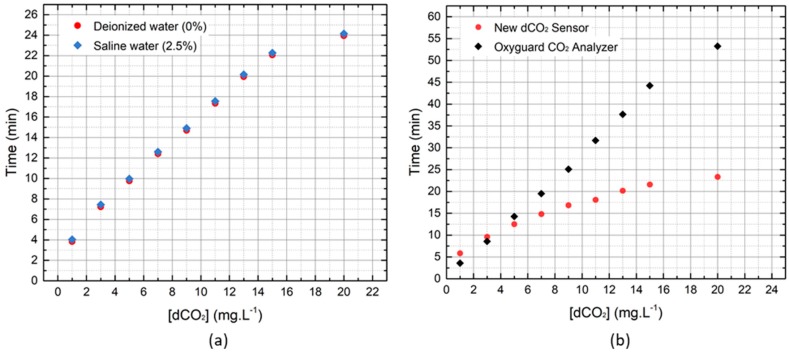
(**a**) Cumulative response time (*t*_95%_) of the new dCO_2_ sensor in different types of water and (**b**) comparison of the cumulative response time (*t*_95%_) between the new dCO_2_ sensor and OxyGuard CO_2_ Analyzer in deionized water.

The response time (*t*_95%_) of the sensor is almost the same in both types of water, with a slight increase for saline water (2.5%) (Figure 7a). The degassing time (*t_g_*) recorded was about twice as long as the response time observed in the reaction to CO_2_ increase (*t_g_* = 2*t*_95%_). Comparing the dCO_2_ optrode and the commercial sensor behavior, in Figure 7b, it is possible to see that the time the new sensor takes to respond to concentration increments of 1.00 mg·L^−1^ and 2.00 mg·L^−1^ decreases with increasing concentration, but it is on average around *t*_95%_ ≈ 3 min. On the other hand, the response time of the commercial sensor increases along the experiment. With this test, it is concluded that the dCO_2_ optrode is faster than the tested commercial sensor in the range of 2.99 to 19.8 mg·L^−1^.

### 3.4. Optrode Sensitivity

The new optrode showed a suitable sensitivity response to small increments of dCO_2_ concentrations in deionized water, as displayed in Table 3. The accuracy increased with increasing dCO_2_ concentrations. The response time in this range is similar to the results observed in higher concentrations.

### 3.5. Dissolved CO_2_ Dynamics in a SRS+RAS Prototype

**Figure 8:**
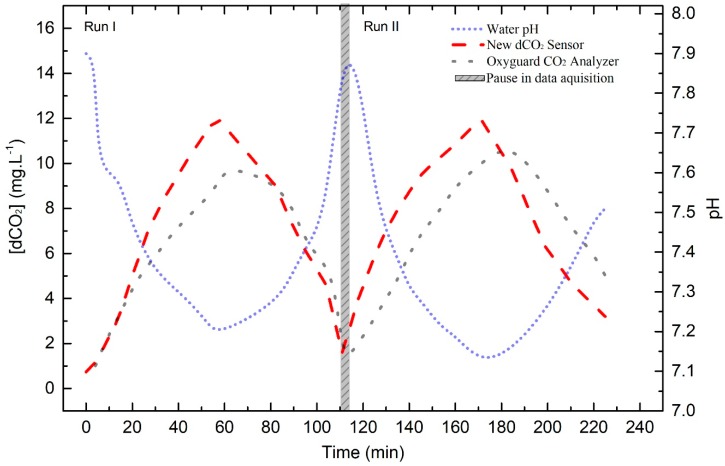
Continuous dissolved CO_2_ profile in the recirculating shallow raceway system (SRS+RAS) prototype, using the new dCO_2_ sensor (dashed line) and the OxyGuard CO_2_ Analyzer (dotted line), and concomitant water pH variations (short dot line).

The goal of this experiment was to simulate the conditions present in fish farms after the feeding events, where it was supposed to have the hardest conditions for monitoring. The results of a monitoring experiment with a duration of 4 h and 15 min (with two complete cycles of gassing and degassing) can be seen in Figure 8. It is visible that both sensors showed the pre-existence of some dissolved CO_2_ in the system water before the gaseous CO_2_ injection. The new sensor showed a starting concentration around 0.75 mg·L^−1^, and OxyGuard showed a dCO_2_ concentration around 1.00 mg·L^−1^ (but the displayed value varied successively between 1.00 and 2.00 mg·L^−1^ before the gas bottle opening). In run 1, after 10 min of CO_2_ injection, the commercial sensor showed a concentration around 3.75 mg·L^−1^, while the dCO_2_ optrode only detected 2.00 mg·L^−1^. This can be explained by the higher initial value displayed by the OxyGuard sensor. However, after 20 min, the values given by both sensors were the same (around 5.00 mg·L^−1^), and from this point on, the new optrode was able to reach the next concentration in a shorter period of time. Furthermore, the discrepancy between both sensors at the same instant should be related to the different response time. The same was observed in the monitoring of the degassing process after the CO_2_ supply was cut (around 60 min).

In run 2 (1 h 50 min after the first one), the dCO_2_ optrode showed a starting concentration of 1.58 mg·L^−1^, and the OxyGuard sensor showed again variations between 1.00 and 2.00 mg·L^−1^. When the CO_2_ supply bottle was opened, the new sensor response was always faster than the used commercial sensor, achieving 4.00 mg·L^−1^ in 10 min, while the Oxyguard sensor only displayed 2.00 mg·L^−1^. Even in the degassing period (started at 170 min), the dCO_2_ optrode showed around 6.00 mg·L^−1^ after 30 min, while the OxyGuard sensor showed 9.00 mg·L^−1^. The two sensors showed again different maximum concentrations. Nevertheless, the dCO_2_ optrode showed the same maximum concentration 1 h after injection in both assays, registering 12.00 mg·L^−1^, while the commercial sensor registered 10.00 and 11.00 mg·L^−1^, respectively. Looking for these results, we can say that the dCO_2_ optrode has shown a better performance than the used commercial sensor, mainly considering the response time. The pH was also monitored, and 10 min after the first CO_2_ injection, it decreased from 7.90 to 7.60, reaching 7.19 in the maximum CO_2_ registered concentration. In run 2, the water pH also started around 7.90 and attained 7.12 in the same time period, which is a result that can be considered a match between both runs. The experimental error for tests performed using the new sensor was around [dCO_2_] ± 0.50 mg·L^−1^. The error was calculated using Equation (7), where *S_y/x_* is the standard error of the estimate, (*y_i_* − *y_i_*′) is the deviation of each measurement, and *n* is the number of points of the calibration curve; and Equation (8), where *S_x_*_0_ is the x error, *y*_0_ is the experimental signal of the instrument for which *x*_0_ is to be determined, and *m* is the number of repetitions [31]. The OxyGuard Analyzer experimental error was given by the manufacturers in the data sheet of the equipment.

(7) $$S_{y/x}=\sqrt{\frac{\sum_{i}{\left(y_{i}-{y_{i}}^{\prime }\right)}^{2}}{n-2}}$$

(8) $$S_{x0}=\pm \frac{S_{y/x}}{b}\times \left(\sqrt{\frac{1}{m}+\frac{1}{n}+\frac{{\left(y_{0}-\bar{y}\right)}^{2}}{b^{2}\sum_{i}{\left(x_{i}-{\bar{x}}_{i}\right)}^{2}}}\right)$$

### 3.6. Laboratory Fish Culture System dCO_2_ Evaluation

**Figure 9:**
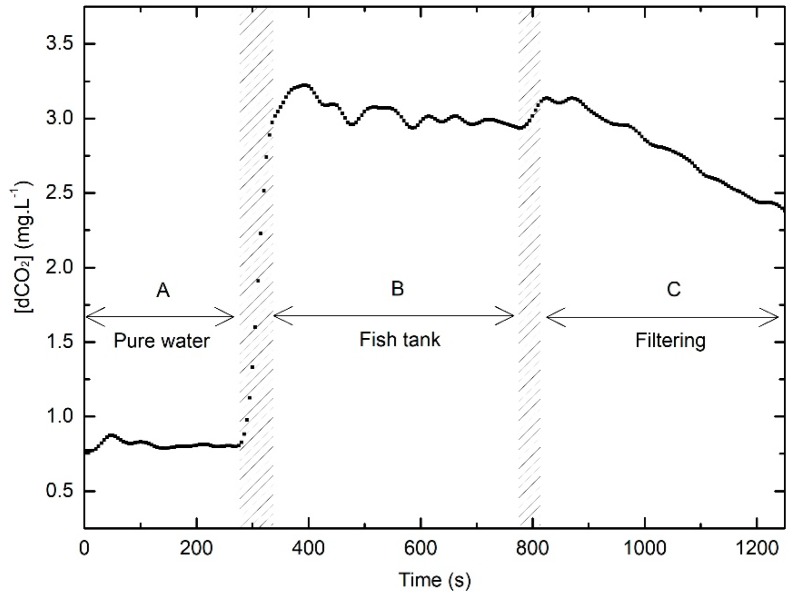
New optrode fish culture system experimental results obtained measuring dCO_2_ produced by rainbow trout respiration in fresh water.

In this test, it was planned to study the performance of the dCO_2_ optrode in a real aquaculture situation with fish in the system and CO_2_ produced by fish respiration. The resultant data are shown in Figure 9. The dissolved CO_2_ optrode and Oxyguard CO_2_ Analyzer^®^ were placed close to each other in the sampling locations, and the samples were taken at the same time. The data logging results showed that the optrode was sensitive to small amounts of dissolved CO_2_ and the readings took around 2 min and 30 s to stabilize, reaching 3.00 mg·L^−1^ (Figure 9B). Simultaneous commercial sensor readings did not change from 1.00 mg·L^−1^ during the experiment. After about 10 min of reading, the sampling point was changed, as the sensors were placed in the biofilter tank (logging was stopped during this switch). As expected, the dCO_2_ optrode showed a decrease in the dissolved gas concentration, reaching 2.35 mg·L^−1^ (Figure 9C). Theoretically, these are acceptable values for dCO_2_ concentrations in those situations, as some degassing occurs in the sump, prior to the biofilter. The commercial sensor maintained the 1.00 mg·L^−1^ reading. Although there was some curiosity of the fish by the new sensor presence (when it is on, a light is seen), no noticeable behavioral changes were observed.

## 4. Discussion

The response of the new dCO_2_ optrode was studied with promising results for real-time monitoring applications. Using the reaction of the sodium carbonate in acid media [32], it was possible to produce known and precise amounts of dissolved carbon dioxide to calibrate the new optrode in the required range of operation. Moreover, the sensor could work in a larger range of dCO_2_ levels with the respective calibration. This method shows that this sensor is capable of operating in environments where the pH is quite low (pH ≤ 3) in contrast with most other naked optical CO_2_ sensors [1].

This type of calibration was also used by Fritzsche et al. (2018) [35] to calibrate a sensor for marine applications. However, at low CO_2_ concentrations, the sensor had a response time of over 30 min. which is not suitable for applications in aquaculture, in contrast with the new optrode (response in the range of ≈120 s for a small variation of concentration ≈0.25 mg·L^−1^). Sensitivity studies showed that the new optrode is capable of detecting and evaluating small dCO_2_ variations, not just in a low range but also in higher dCO_2_ concentrations. Furthermore, the sensitivity of the membrane is not affected by changing the work environment. In other works [1], the change from air to water causes a decrease in the CO_2_ sensitivity, which is not observable in the results presented for the new optrode. The precision of the new optrode was also attested, showing a better performance in saline water (2.5%), especially for higher concentrations (>10 mg·L^−1^). Although the precision reported by Borges et al. [14] for the commercial sensor in saline water is similar, the new dCO_2_ optrode shows superior accuracy in the experimental tests. In addition, in what concerns shelf life, the sensing film can be stored in a sodium carbonate solution [36] at low temperatures (2 < T < 10 °C) for at least 6 months, and can be used after a new calibration. As stirring is not needed, the sensor is suitable to be used for continuous dissolved CO_2_ monitoring, contrary to the systems used by other authors [14,37].

Although the sensor body is 3D printed, it was proved that this material resulted in a robust enough apparatus that can be used in real aquaculture environment, as SRS+RAS systems. In addition, its size is appropriate to be used in such working conditions. The electronic part needs to be connected to a PC, which acts as an energy supplier and also allows interaction with the specially designed control software, which processes and stores the data in the memory with a minimum acquisition time of 100 ms. Overall, it was demonstrated a compact portable solution that could be used to realize multipoint readings in different parts of the recirculating system. In future embodiments, this configuration can become even more compact and robust using more durable materials and a micro-controlled electronics module with wireless capabilities.

## 5. Conclusions

A new dissolved carbon dioxide sensing platform was demonstrated and validated, showing its suitability and precision for continuous direct measurements of dissolved CO_2_ concentrations in different aqueous solutions. In general, the best performance was found in saline solutions following by the deionized water solutions. Sensor validation in the SRS+RAS prototype and in the laboratory fish culture showed its versatility and appropriateness to be used in aquaculture systems as well as in a larger field of applications.

## Figures and Tables

**Table 1:** Calibration data obtained for the new optrode chemical calibration and the respective limit of detection (LOD) and limit of quantification (LOQ).

Calibration y = a + bx	Intercept (a)	Slope (b)	Pearson’s	R-Square	Adj. R-Square	LOD (mg·L^−1^)	LOQ (mg·L^−1^)
**Deionized water**	0.666 ± 0.008	0.271 ± 0.009	0.996	0.992	0.991	1.225	1.843
**Saline water (2.5%)**	0.501 ± 0.013	0.405 ± 0.014	0.996	0.992	0.991	1.048	1.154

**Table 2:** Optrode performance (mean ± standard deviation; n = 3) in different aqueous solutions at different dCO_2_ concentrations. CV: coefficient of variation.

[dCO_2_] (mg·L^−1^)	Deionized Water (0%)			Saline Water (2.5%)		
	M.C. ^1^ (mg·L^−1^)	P ^2^ (CV%)	|Acc| ^3^ (mg·L^−1^)	M.C. (mg·L^−1^)	P (CV%)	|Acc| (mg·L^−1^)
**1.00**	1.14 ± 0.10	5.87	0.14 ± 0.07	1.13 ± 0.01	1.19	0.13 ± 0.01
**2.99**	2.77 ± 0.14	5.15	0.08 ± 0.05	2.81 ± 0.03	1.00	0.06 ± 0.01
**4.99**	4.56 ± 0.34	7.52	0.09 ± 0.07	4.58 ± 0.07	1.50	0.08 ± 0.01
**6.98**	6.47 ± 0.62	9.56	0.09 ± 0.06	6.49 ± 0.09	1.51	0.07 ± 0.01
**8.97**	8.61 ± 0.88	10.2	0.09 ± 0.03	8.41 ± 0.11	1.31	0.06 ± 0.01
**10.9**	11.1 ± 1.7	15.1	0.11 ± 0.10	10.7 ± 0.2	2.70	0.02 ± 0.02
**12.9**	13.5 ± 2.2	15.9	0.11 ± 0.11	13.1 ± 0.2	1.51	0.02 ± 0.01
**14.9**	16.1 ± 3.2	19.8	0.11 ± 0.11	15.6 ± 0.4	2.62	0.05 ± 0.03
**19.8**	22.2 ± 4.2	19.1	0.14 ± 0.19	22.6 ± 0.3	1.43	0.14 ± 0.02

^1^ measured concentration; ^2^ precision; ^3^ accuracy (absolute values).

**Table 3:** Sensitivity of the new dCO_2_ sensor to 0.25 mg·L^−1^ increments (starting from 1.00 mg·L^−1^) until signal time stabilization to 95% of equilibrium response (*t*_95%_) in deionized water.

[dCO_2_]_final_ (mg·L^−1^)	M.C. ^1^ (mg·L^−1^)	|Acc| ^2^ (mg·L^−1^)	Response Time (s)
**1.25**	1.39	0.56	103
**1.50**	1.55	0.11	152
**2.75**	1.76	0.01	137

^1^ measured concentration; ^2^ accuracy (absolute values).
